# Social capital – a mixed blessing for women? A cross-sectional study of different forms of social relations and self-rated depression in Moscow

**DOI:** 10.1186/s40359-016-0144-1

**Published:** 2016-07-22

**Authors:** Sara Ferlander, Andrew Stickley, Olga Kislitsyna, Tanya Jukkala, Per Carlson, Ilkka Henrik Mäkinen

**Affiliations:** Stockholm Centre for Health and Social Change (SCOHOST), Department of Sociology, School of Social Sciences, Södertörn University, 141 89 Huddinge, Sweden; European Centre on Health of Societies in Transition (ECOHOST), London School of Hygiene and Tropical Medicine, London, UK; Department of Human Ecology, Graduate School of Medicine, University of Tokyo, Tokyo, Japan; Department of Quality of Life Measurement Problems at the Institute of Economics, Russian Academy of Sciences, Moscow, Russia; Stockholm Centre for Health and Social Change (SCOHOST), Department of Social Work, School of Social Sciences, Södertörn University, Huddinge, Sweden; Department of Sociology, Uppsala University, Uppsala, Sweden

## Abstract

**Background:**

Depression is a major health problem worldwide, especially among women. The condition has been related to a number of factors, such as alcohol consumption, economic situation and, more recently, to social capital. However, there have been relatively few studies about the social capital-depression relationship in Eastern Europe. This paper aims to fill this gap by examining the association between different forms of social capital and self-rated depression in Moscow. Differences between men and women will also be examined, with a special focus on women.

**Methods:**

Data was obtained from the Moscow Health Survey, which was conducted in 2004 with 1190 Muscovites aged 18 years or above. For depression, a single-item self-reported measure was used. Social capital was operationalised through five questions about different forms of social relations. Logistic regression analysis was undertaken to estimate the association between social capital and self-rated depression, separately for men and women.

**Results:**

More women (48 %) than men (36 %) reported that they had felt depressed during the last year. An association was found between social capital and reported depression only among women. Women who were divorced or widowed or who had little contact with relatives had higher odds of reporting depression than those with more family contact. Women who regularly engaged with people from different age groups outside of their families were also more likely to report depression than those with less regular contact.

**Conclusions:**

Social capital can be a mixed blessing for women. Different forms of social relations can lead to different health outcomes, both positive and negative. Although the family is important for women’s mental health in Moscow, extra-familial relations across age groups can be mentally distressing. This suggests that even though social capital can be a valuable resource for mental health, some of its forms can be mentally deleterious to maintain, especially for women. More research is needed on both sides to social capital. A special focus should be placed on bridging social relations among women in order to better understand the complex association between social capital and depression in Russia and elsewhere.

## Background

Depression is a common mental disorder that is characterised by “sadness, loss of interest or pleasure, feelings of guilt or low self-worth, disturbed sleep or appetite, feelings of tiredness, and poor concentration” [1]. According to the World Health Organisation (WHO) [2], depression is the third leading contributor to the global burden of disease and is anticipated to become the highest contributing factor by 2030. Nevertheless, for women, depression is already the leading cause of disease worldwide [2]. Significant gender differences have been found in many countries, with depression being about twice as common among women than among men [3, 4].

In low- and middle-income countries, among which most Eastern European countries are included, depression is also the leading cause of the global disease burden [2]. In their study of 23 countries, Van de Velde et al. [4] found that the prevalence of depression was highest in the Eastern and Central European countries. In Russia, however, relatively little is known about depression [5]. Nonetheless, some studies have shown that levels of depression are high in Russia [6], and especially among women [7]. For example, in a study in Novosibirsk in 1999–2000, depression was reported by 23 % of men and 44 % of women [8]. During the same years, in Arkhangelsk, depression, anxiety and/or sleeping disorders affected 33 % of men and 69 % of women [9]. Consequently, there is a significant gender difference in depression in Russia.

Depression has been related to a number of factors, such as alcohol consumption [10], economic situation [8] and social capital [11]. However, as recently stated by Levecque and Van Rossem: “Although depression is widespread, the complex mechanisms causing depression are still not clearly understood” ([12], p. 50). Among the causes of depression, social capital may be of particular importance [13]. The association between social capital and depression is often traced back to the work of Emile Durkheim, who found a link between social integration and suicide rates in different societies [14]. In the WHO report *Promoting Mental Health*, social capital is suggested as one of the factors that might promote better mental health [15]. Unfortunately, despite the potential importance of the social capital-mental health relationship, relatively few studies have been conducted on this topic in Eastern Europe [16]. Although there have been reports on the prevalence of depression in Russia, few studies have examined how this disorder affects each gender [5, 17]. Women have generally been overlooked in health studies in Russia, as men have suffered the heaviest burden of mortality [18]. The aim of this article therefore is to fill these research gaps by studying the association between different forms of social capital and self-rated depression in Moscow. Women and men will be analysed separately, with a special focus on women.

### Social capital

Social capital is often described as a valuable resource accessed through social relations. Bourdieu defines it as “the actual or potential resources which are linked to possession of a durable network of more or less institutionalised relationships of mutual acquaintances and recognition – or in other words, to membership of a group” ([19], p. 248). Putnam writes that “the core idea of social capital is that social networks have a value” ([20], p. 18). Coleman also views social capital as a valuable resource, but acknowledges that “a given form of social capital that is valuable in facilitating certain actions may be useless or even harmful for others” ([21], p. 98). This argument supports the view that there can be a negative side to social capital [22].

### Different forms of social capital

Although social capital has been defined in various ways, most definitions include two aspects: one structural and the other cognitive, i.e. the social relations themselves and their more qualitative aspects, such as trust and reciprocity. Structural social capital is often divided into informal and formal forms [21, 23]. The former comprises casual relations with family and friends, whereas the latter involves more rule-bound networks, such as voluntary associations. Among informal contacts, a further distinction can be made between relations within and outside of one’s family. Family is often viewed as the main form of social capital [24, 25], i.e. family-based social capital. Family has been defined as both immediate family and extended family (the latter e.g. relatives) [26]. As put by Astone et al.: “Family behaviours, including marriage and childrearing, remain the classic examples of investment in social capital” ([27], p. 18). More recent sociological research has also stressed the importance of family as social capital [28]. In contemporary society, however, social relations often extend beyond family. People have access to a variety of relationships: a few family relations and perhaps hundreds of peripheral ones [29].

Whereas Bourdieu and Coleman focused on strong familial ties, Putnam, especially in his early work, focused on the weaker, more formal ties outside the family that, for instance, can be accessed via voluntary associations [23]. Weak ties refer to relations among people who are distant from each other, such as acquaintances [30]. A more recent distinction is the one between bonding and bridging social capital [31]. Bonding relations are homogenous in terms of certain social characteristics, such as age or educational level, whereas bridging relations are heterogeneous and link people across different groups, such as intergenerational relations. Although these distinctions are related, they are not synonymous. Strong ties exist between people who are emotionally close, bonding ties between people who are similar. Weak ties unite emotionally distant people, whereas bridging ties connect people who are different from each other. For a more detailed discussion of the different forms of social capital, see Ferlander [32].

### Social capital and mental health

There is consistent evidence linking social capital to physical health, but fewer studies have linked social capital with mental health [33]. Though they may not have used the term social capital, many earlier studies found a positive link between strong family ties and mental health [34, 35]. It has been shown, for example, that married individuals exhibit fewer depressive symptoms than non-married individuals [36]. Marriage generally has a positive effect on well-being through the exchange of emotional support and increased economic well-being [37]. More extended family, i.e. relatives, play an important role in terms of social support [38]. Marriage and other family relations are vital buffers against stress [39]. Strong and bonding relations, however, can also be a source of strain, leading to feelings of obligation and poor health [22, 40].

In a study in a low-income area of the US, Mitchell and LaGory found that bonding social capital increased mental distress, whereas bridging contacts decreased it [41]. The authors concluded that the obligations of bonding social capital might be a burden and a source of stress for people living in economically deprived areas. Similarly Caughy and colleagues [42] found that higher levels of social capital among parents were related to higher levels of depression among children in poor areas. In wealthy areas, however, higher parental social capital was associated with better mental health in children. Thus, the social capital-mental health link varies not only between different *forms* of social capital, but also between different *groups.* In relation to this, many scholars have stressed the importance of comparing different groups when studying the association between social capital and health [43]. Vyncke et al. ([44], p. 960), for instance, recently wrote that: “Future studies should seek to identify subgroups for whom social capital might be particularly influential, by transcending ‘simple’ dyads such as ‘men versus women’”.

### Social capital, gender and mental health

Gender differences have received relatively little attention in social capital research [44]. It has been found, though, that women tend to be more family-oriented, often occupying the role of “kin-keeper” in the family [45]. Spending more time performing this role, women might socialise less outside the family, as shown by the observation that women belong to fewer voluntary associations than men [46]. However, women tend to bear the cost of creating social capital, while deriving fewer benefits from it than men [45]. Although generally ignoring gender issues in relation to social capital, Bourdieu argues that women enable men to accumulate social capital through social activities, such as the exchange of gifts and telephone calls [47]. A recent study in Russia gives an example of this by showing that women spend more time providing unpaid assistance than men, even though they face a greater risk of nonreciprocation [48].

It has also been claimed that women do not receive the same health benefits from their contacts as their male counterparts. A number of studies have found a positive link between social capital and self-rated health among men, but not among women [e.g. 49, 50]. Pertaining to depression, nevertheless, social relations seem to have a stronger effect on women [33, 51]. In their classic work, Brown and Harris found that women with a close confidant were less likely to become depressed during traumatic life events [52]. There is also some evidence that the effect of divorce in terms of depression is greater for women than for men [53]. Similar findings have also been reported for Eastern Europe, for instance in Ukraine where it was recently shown that divorce and widowhood are associated with female depression [54].

In contrast, social relations may also *increase* levels of mental illness among women with fewer economic resources. Kawachi and Berkman argue that differences in gender support may partly account for the higher prevalence of psychological distress among women compared to men, particularly if social relations involve strain associated with obligations to provide support for others [55]. In two different studies of mothers in low-income settings, social capital was associated with a higher risk of mental health problems [56, 57]. The authors hypothesised that participating in many social activities may have placed an additional burden on already overextended mothers. Similar effects have been found among mothers in Russia [58, 59].

### Social capital in Russia

Russia is often described as being characterised by a weak civil society and low levels of institutional trust [60, 61]. As a large number of the social safety nets that were available in the Soviet period, such as childcare and maternity benefits, either weakened or disappeared after the collapse of communism [62, 63], many Russians, particularly women, have turned to their informal contacts for social and emotional support [64]. Family and friends are argued to be vital forms of social capital in Eastern Europe [65], and in Russia, it has been suggested that the “family may be the only island of stability in the boundless ocean of uncertainty” ([66], p. 367).

In most Russian families, the link between generations is strong. For instance, studies have shown that relations between daughters and mothers in Russia are very amicable [67]. Women’s family relations often involve exchanges of emotional support across generations, but they can also be fraught with hierarchical and internal power relations, particularly between women of different ages [58, 59]. This ambivalence towards intergenerational relations is also shown by Minnigaleeva and her colleagues, who found that the general view of the elderly *outside* one’s family in Russia is negative—they are often described as poor, passive and unable to adapt to modern life [68]. However, when people speak about the elderly *within* their family, the image is more positive, with the elderly being described as “active, kind, wise and caring” ([68], p. 64–65).

In Russia, family may be even more important for women than for men, as women tend to be more economically dependent than men [69]. Although the high levels of female employment present during the Soviet period have persisted, women’s position in the labour market has deteriorated as a result of gender discrimination [70]. For example, a recent national report showed that the ratio of female to male earnings was 65 % [71]. Single women are thus at risk of living in poverty [17]. Moreover, conservative attitudes suggesting that women should return to their ‘traditional’ role in the home have re-emerged [58]. Gender roles are highly traditional in Russia, with women undertaking most of the domestic and child-caring duties [63, 72]. In trying to balance home with work, the ‘double burden’ is heavy for many Russian women [17].

There has been a trend towards smaller families in Russia with decreasing rates of marriage and increasing divorce rates [59, 66]. Attitudes are also beginning to change in Russia, especially among the young and highly educated, who have more liberal attitudes towards gender roles [73]. It has also been argued that there has been an increase in detached relations, i.e. relations with low levels of emotional closeness, among Russian women [67]. Among divorced Muscovites, there is a high prevalence of loneliness, and more women than men report that they often feel lonely [74]. This might have negative health effects, as in a society with weak social safety nets, the most exposed groups are probably those without strong ties. Indeed, it has been hypothesised that social capital might be particularly strongly related to mental health in these types of societies [33]. The need for more studies on the social capital-depression link in low- and middle-income countries, such as Russia, has also recently been emphasised [13].

## Methods

The data used in this study came from the Moscow Health Survey, which was conducted in 2004. The survey aimed to study self-rated health in Moscow in relation to social and economic factors. As earlier research had indicated that social capital might be an important factor in the health of Russians [75], the survey explicitly incorporated a range of social capital measures. The use of this dataset in the current study thus makes it possible to explore the association between different forms of social capital and self-reported depression in a representative sample of the population in the largest city in Eastern Europe - where social capital might be especially important given the social and economic turmoil that has characterised post-Soviet Russia for a long period [60–64]. Indeed, according to Rose [75], Russia is especially suitable for studying social capital and health, as the collapse of the Soviet Union was far more pervasive in its effects than the social crisis Durkheim, in his time, referred to as causing anomie and suicide [14].

A gender- and age-stratified random sampling method was used across the 125 municipal districts of Greater Moscow, where the city telephone network formed the sampling frame (nearly all of Moscow’s flats had a telephone in 2004). Face-to-face interviews were done by trained interviewers with Muscovites aged 18 and above based upon a structured questionnaire. The final sample consisted of 1190 individuals with a response rate of 47 %. Fifty-seven percent of the respondents were women. The average age of the sample was 47 years. More than half of the respondents (53 %) had a high level of education, whereas one-fifth (19 %) had a low educational level. Thirty-six percent of the sample had many (2 or more) economic problems. Except for an over-representation of the highly educated, the sample was generally representative of Moscow’s population. The gender and age distributions closely mirrored those of Greater Moscow as a whole. For a more detailed discussion of the survey methodology, see Vågerö and colleagues [76].

### Variables

Information on self-rated depression, the dependent variable, was obtained by asking: ‘During the last 12 months, have you had any of the following health problems? If yes, were they severe or mild?’ One of the response categories was ‘nervous disorders, depression.’ The answers were then dichotomised into ‘depression‘ (severe or mild) and ‘no depression’*.* Single-item measures of self-rated mental health are increasingly being used in health research and population health surveys, as they reduce the burden for the respondents compared to longer scales [77]. Similar measures of depression have been used previously in Russia [9] and elsewhere. For example, in a study of 29 countries using *World Value Survey* data, the question, ‘During the past weeks, did you ever feel… Depressed or very unhappy?’ was used to assess depression [3].

Five indicators of structural social capital were used as independent variables. Following previous studies, for instance by Coleman [25], Helliwell and Putnam [78], and Furstenberg [79], marital status and the frequency of visiting relatives were used as indicators of family-based social capital. Marital status was divided into three categories: ‘married or cohabiting’, ‘divorced or widowed’ and ‘never married’. Contact with relatives was measured by the question, ‘Do you tend to visit relatives?’ There were three response categories—‘often, rarely or never’—which were recoded into two categories: ‘regular’ (often) and ‘little (rarely/never) contact’.

The other social capital indicators focused on extra-familial relations: contacts with friends and acquaintances, age-bridging contacts with people from different age groups and membership in voluntary associations. The first two were measured with the following questions: ‘Do you tend to visit friends and acquaintances?’, which is a common indicator of social capital [80], and ‘How often do you mix with people from different age groups (outside the family)?’ There were three response categories—‘often, rarely or never’—which were recoded into two categories: ‘regular’ (often) and ‘little (rarely/never) contact’. In addition to measuring social capital outside the family, the first question also measures both strong (friends) and weak (acquaintances) ties [30], whereas the second, following Mitchell and LaGory’s [41] study, measures bridging relations in terms of contact with people from different age groups outside the family. In this study, the latter is labelled age-bridging contacts, as age is the cross-cutting factor in focus.

Membership in a voluntary association measures a more formal type of social capital. It is probably the most common indicator of social capital in general including in mental health studies [81]. Associational membership was measured with the question: ‘Are you a member of any of the following organisations or associations: a) sports club, b) environmental organisation, c) cultural, musical, dance or theatre society, d) women’s organisation, e) temperance organisation, f) local action group, g) political party, h) trade union, i) business or employer’s organisation, j) religious organisation, k) other club or association?’ There were three response categories —‘yes, active member’, ‘yes, ordinary member’ and ‘no’—which were recoded into ‘member’ (active or ordinary member of at least one voluntary association) and ‘non-member’.

As in other studies about social capital and health [16, 49], demographic (age) and socioeconomic (educational level and economic situation) variables were included in the analysis. Educational level was divided into three groups: ‘high’ (higher or incomplete higher), ‘medium’ (specialised secondary or vocational technical school) and ‘low’ (common secondary or less). To assess the economic situation, the respondents were asked whether, during the previous twelve months, their family ‘had to rely on outside help to pay regular expenses on time (e.g., rent)’, ‘could not have meat or fish more than once or twice a week’, ‘had to refrain from purchasing necessary clothes or footwear’, and ‘involuntarily had to refrain from taking part in social or cultural activities, such as going to a restaurant, cinema, theatre, etc.?’ The answers were added to create a scale from 0–4, which was further divided into two groups: those experiencing ‘few’ (0–1) and ‘many’ (2 or more) types of economic problem.

### Statistical analysis

First, descriptive statistics were calculated to determine the levels of depression and social capital in Moscow (Table 1). Women and men were compared using a chi-square analysis. Logistic regression analysis was then undertaken to estimate the association between different forms of social capital and self-rated depression separately for men and women, while controlling for the effects of age, educational level and economic problems (Table 2). There were two regression models: in Model 1, the association between each variable and reported depression was separately examined while adjusting only for age; in Model 2, the association was examined while adjusting for all the other variables in the model.

**Table 1:** Descriptive statistics of self-rated depression and social capital among respondents aged 18 and over in Moscow, 2004, by gender (%)

Variable	Men (*n* = 510)	Women (*n* = 680)	Total (*n* = 1190)	p*
Self-rated depression				
Severe	25	32	29	0.000
Mild	11	16	14	
None	64	52	57	
Family relations				
Marital status				
Married/cohabiting	66	50	57	0.000
Divorced/widowed	13	35	25	
Never married	21	15	18	
Contact with relatives				
Regular	42	43	42	0.866
Little	58	57	58	
Extra-familial relations				
Contact with friends/acquaintances				
Regular	52	46	48	0.039
Little	48	54	52	
Age-bridging contacts				
Regular	58	53	55	0.078
Little	42	47	45	
Voluntary associations				
Member	30	22	25	0.002
Non-member	70	78	75	

* *p* for gender difference

**Table 2:** Self-rated depression among respondents aged 18 and over in Moscow. Odds ratios (OR) with 95 % confidence intervals (CI) estimated from binary logistic regression. Model 1: age adjusted; Model 2: mutually adjusted

Variables	Men (*n* = 507)	Women (*n* = 678)						
	Model 1	Model 2	Model 1	Model 2				
	OR	95 % CI	OR	95 % CI	OR	95 % CI	OR	95 % CI
Age	1.00	0.99–1.01	0.99	0.98–1.00	1.00	0.99–1.01	1.00	0.99–1.01
Educational level								
High	1.00		1.00		1.00		1.00	
Medium	0.89	0.58–1.36	0.78	0.49–1.22	0.91	0.64–1.29	0.85	0.59–1.22
Low	1.04	0.66–1.65	1.02	0.63–1.65	0.75	0.50–1.12	0.74	0.49–1.12
Economic problems								
Few	1.00		1.00		1.00		1.00	
Many	2.34	1.57–3.51***	2.49	1.65–3.78***	1.48	1.09–2.01*	1.52	1.11–2.07**
Family relations								
Marital status								
Married/cohabiting	1.00		1.00		1.00		1.00	
Divorced/widowed	1.20	0.70–2.07	1.13	0.64–1.98	1.48	1.02–2.14*	1.49	1.03–2.17*
Never married	1.03	0.60–1.76	1.10	0.62–1.95	0.96	0.59–1.56	0.94	0.56–1.55
Contact with relatives								
Regular	1.00		1.00		1.00		1.00	
Little	1.62	1.10–2.39*	1.51	1.00–2.30	1.45	1.06–1.98*	1.57	1.10–2.24*
Extra-familial relations								
Contact with friends/ acquaintances								
Regular	1.00		1.00		1.00		1.00	
Little	1.63	1.08–2.45*	1.44	0.93–2.25	1.12	0.81–1.55	0.97	0.67–1.40
Age-bridging contacts								
Regular	1.00		1.00		1.00		1.00	
Little	0.99	0.67–1.47	0.88	0.58–1.33	0.72	0.52–0.98*	0.72	0.51–1.00*
Voluntary associations								
Member	1.00		1.00		1.00		1.00	
Non-member	1.07	0.70–1.63	1.07	0.68–1.68	0.96	0.66–1.40	1.04	0.70–1.54

*p < 0.05 **p < 0.005 ***p < 0.001

NOTE: Standardised according to the educational-level proportions of the Moscow City population given in the 2002 census for the population over 18 years

The results of these analyses led to a further analysis of the association between age-bridging contacts outside the family and women’s reported depression (Table 3). Logistic regression analyses between these variables were performed for subgroups divided according to age, educational level, economic situation, marital status, presence of small children and the nature of their work, controlling for all other variables. The results are presented as odds ratios (OR) with 95 % confidence intervals (CI) and *p*-values. The level of statistical significance was set at *p* < 0.05, with statistically significant *p*-values presented with at least one asterisk (*).

**Table 3:** Separate logistic regression analyses between age-bridging contacts and women’s self-rated depression for various subgroups within the sample. Odds ratios (OR) with 95 % confidence intervals (CI)

	OR	95 % CI	*p*	n	Nagelkerke
Age					
18–40 years	0.45	0.26–0.80**	0.006	239	0.057
41–94 years	0.90	0.61–1.32	0.582	441	0.002
Educational level					
High	0.55	0.33–0.92**	0.021	341	0.027
Medium	1.14	0.67–1.95	0.628	215	0.002
Low	0.61	0.31–1.19	0.144	124	0.022
Economic problems					
Few	0.50	0.31–0.81*	0.005	295	0.052
Many	0.98	0.64–1.50	0.915	385	0.012
Marital status					
Married/cohabiting	0.75	0.49–1.17	0.210	337	0.006
Divorced/widowed	0.80	0.46–1.40	0.438	236	0.009
Never married	0.44	0.18–1.08	0.072	105	0.045
Small children					
Children 0–5 years	0.21	0.06–0.68*	0.010	63	0.217
No small children	0.80	0.58–1.12	0.191	617	0.004
Nature of work					
Manual labour	0.88	0.37–2.08	0.774	84	0.001
Manual labour, skilled	0.50	0.19–1.27	0.145	76	0.039
Intellectual work	0.82	0.43–1.57	0.547	141	0.004
Intellectual work, skilled	0.57	0.32–1.02	0.056	253	0.035
Intellectual work, leading position	1.07	0.21–5.43	0.931	41	0.031

*p < 0.05 **p < 0.005 ***p < 0.001

NOTE: Standardised according to the educational-level proportions of the Moscow City population given in the 2002 census for the population over 18 years

To overcome the potential problem of over-representation of the highly educated in the analysis, the data were weighted in order to match the educational distribution given in the All-Russia Population Census 2002 for Moscow city [82]. Proportional weights were calculated for the three educational groups and separately for men and women. See also the study by Jukkala et al. [83]. Finally, to examine the validity of the dependent variable, an additional analysis was undertaken where the association between self-rated depression and other aspects of major depressive disorder (i.e. insomnia and problematic weight loss) was examined through a chi-square test.

## Results

### Descriptive results

Table 1 shows that more than two-fifths (43 %) of the sample reported that they had felt depressed during the last twelve months. Twenty-nine percent reported that they had experienced severe depression. A chi-square analysis showed that the prevalence of self-rated depression (severe and mild) was significantly higher among women (48 %) than among men (36 %). Nearly one-third (32 %) of all women in Moscow reported that they had felt severely depressed.

Regarding family-based social capital (i.e. family relations; Table 1), 57 % of the sample was married or cohabiting and more than two-fifths (42 %) visited relatives regularly. In terms of extra-familial relations, almost half (48 %) of the respondents visited friends or acquaintances regularly and more than half (55 %) regularly mixed with people from other age groups outside the family (i.e. age-bridging contacts). A quarter of the sample (25 %) were members of at least one voluntary association. There were also some statistically significant gender differences in social capital. Two-thirds of the men in the sample were married or cohabiting (66 %), compared to only half of the women (50 %). Approximately half of the men (52 %), as compared to less than half of women (46 %), maintained regular contact with friends and acquaintances. Thirty percent of men were members of some form of voluntary association, compared to 22 % of women. No statistically significant gender differences were found in contact with relatives and contact with different age groups outside the family (i.e. age-bridging contacts).

### Multivariable results

Table 2 shows that neither age nor educational level was significantly associated with self-rated depression, although economic problems were. In the fully adjusted model, Model 2, the odds of reporting depression among men experiencing many economic problems were more than twice as high compared to those among men with few economic problems (odds ratio (OR) for men = 2.49), whereas the corresponding OR for women was 1.52.

For social capital, a statistically significant association was found between family relations and women’s reported depression in both models. Women who were divorced or widowed had higher odds of reporting depression (OR = 1.49) than those who were married or cohabiting. Women who had little contact with relatives were also more likely to feel depressed than those with more regular contact (OR = 1.57). Concerning extra-familial social relations, there was no association between contact with friends or membership in voluntary associations and self-rated depression for either sex. A statistically significant association was found, however, between age-bridging contacts outside the family and women’s depression in both models. Women who had little contact with people from other age groups outside the family were *less* likely to feel depressed (OR = 0.72) than those who had more regular contact. Hence, age-bridging social capital outside the family seems to increase the risk of reporting depression among women in Moscow.

To examine the above finding further, the regression analysis between age-bridging contacts and women’s reported depression was repeated for a number of groupings within the sample in Table 3. The protective effect of having fewer age-bridging contacts was statistically significant in four subgroups: women 18–40 years old, those with a high educational level, those with few economic problems and those with small (0–5 years) children. The latter was the subgroup that had the strongest effect of age-bridging contacts to reported depression, but that had a lower significance level than the others due to the reduced number of cases in that group. Among the variables with a higher significance level (**), age had the strongest effect. Accordingly, young women and women with small children seem to be especially affected by these forms of social relations.

## Discussion

This study examined the association between social capital and self-rated depression in Moscow, with a special focus on women. In accordance with previous studies in Russia, there was a high level of depression in Moscow [6], especially among women [7]. There were also gender differences in social capital. Women were less likely to be married, have contact with friends or be members of voluntary associations than men [46]. Gender differences in the relationship between social capital and depression were also found. A significant association between the two was found only among women, supporting the idea that, in terms of depression, social capital has a stronger effect on women than on men [51]. For women in Moscow, family relations seem to decrease the risk of depression, whereas contacts across age groups *outside* the family seem to *increase* mental distress. In line with previous family studies in Russia [58], these findings indicate that social capital can constitute a mixed blessing for women in Moscow.

### Family relations and women’s depression

Divorced or widowed women and women who had little contact with their relatives had higher odds of reporting depression than those with more family-based social capital. This finding supports theories stressing that the family is a valuable form of social capital [24, 25], particularly for women [45]. It also supports studies that show that in Russia, where civil society is weak and institutional trust is low [61], the family is a significant form of social capital [66].

The importance of the family for mental health has been stressed in a number of previous studies, both in Russia and elsewhere [52]. Marriage and kin relations provide resources in the form of emotional, instrumental and social support [37, 38], such as having someone to talk to about problems, which can reduce stress [39]. In Russia, studies have found that relations between daughters and mothers are very amicable and that most young married women have regular contact with their mothers for emotional support [67]. The findings are supported by evidence showing that marital status is a stronger predictor of depressive symptoms for women than for men in general [53], as well as in Eastern Europe [54]. This might be especially true in Russia, as the collapse of communism created stressful conditions for women, such as losing access to societal support and being at greater risk of living in poverty as single parents [17, 62]. Also, women in Moscow may rely more on relatives as they have less social capital than men – being less likely to be married and having fewer contacts outside of immediate family members.

### Age-bridging relations and women’s depression

The results of this study also indicate that there can be negative aspects of social capital, as women with regular contacts across age groups outside the family were more likely to report depression than those with less bridging relations. This finding supports arguments that certain forms of social capital might be harmful [21, 22], and especially for certain groups [42]. However, it contradicts more general findings suggesting that bonding social capital is most strongly related to poor mental health [41]. In this study, bridging relations were related to self-rated depression among women.

These findings might be explained by workplace research in general [84] and Bourdieu’s argument [24] that there are generational conflicts over economic and cultural resources. Different work values across generations can lead to conflict. In a study of Russian culture [85], generational differences were found, with people below the age of forty converging more towards Western social values than older generations. In terms of Russian family relations, although women’s networks across generations often involve an exchange of emotional support, there are also elements of unequal power relations, conflict and tensions [58]. According to Utrata [59], who studies Russian single-mother families, there is an intersection between age and gender that produces constraints, with age being the primary organising principle of power. As women in Moscow seem to have less access to social capital than men, it may also be hypothesised that women may have more conflict within the social relations that they have.

If this observation is also mirrored in extra-familial relations, it may help explain our findings, as, through performing their dual role, many women come into contact with numerous extra-familial figures of various ages, through child-care institutions or employers, for example, where inequalities in power can be keenly felt. This inequality might be especially burdening for mothers who are already strained [57–59]. Several studies have found that parents, especially mothers, are more psychologically distressed than non-parents [37]. In Russia, there is marked gender discrimination in the labour market [70], with women, and especially mothers [86], often being discriminated against by employers [87]. Polls among professional women have shown that what could be labelled sexist at a Western workplace is viewed as normal in Russian work relations [88]. There is extensive evidence that bullying and job strain, which is more common among women, is related to depression [89]. Given this, it is possible that many women in Moscow, and elsewhere, experience more mental distress than men due to gender discrimination, conflict and strain from extra-familial ties across generations, leading to feelings of depression.

### Other factors and depression

Neither age nor educational level was significantly related to self-rated depression in Moscow, in accordance with various studies indicating that depression affects individuals across the population [2]. The respondents’ economic situation, though, had a strong link with their reported depression, particularly among men, which confirms findings from previous studies in Russia [6]. Being unable to meet one’s basic needs increased the odds of reporting depression for both genders. Economic problems may be a source of anxiety and mental distress [90], especially in Russia, where many of the social safety nets that were available during the Soviet period have weakened or disappeared [62, 63]. Despite the importance of economy for depression, however, social capital was statistically significantly associated with women’s reported depression in Moscow.

### Methodological limitations

This study has some methodological limitations that should be mentioned. In social research, measurement error is always a possibility. For self-rated depression, as in several other studies [3, 9, 77], a single-item measure was used. Previous research about the reliability and validity of single-item depression measures has found that results can vary between different contexts and populations [91]. However, there has been relatively little research on these measures in the general adult population. A meta-analysis of studies of primary care patients showed that the single-item test had poor sensitivity, correctly identifying less than one-third with depression [92]. A more recent study of chronic pain patients, however, has indicated that these measures can correctly identify most depressed patients [93], while other studies have found high sensitivity but lower specificity [94, 95]. The only large-scale (US) general population study we could locate concluded that a single-item question worked well in detecting depressed adults [96]. However, using previous research to judge the quality of our measure is difficult. Given this, we analysed it in relation to other aspects of major depressive disorder [97]. This showed that respondents who were depressed were also significantly more likely to report suffering from insomnia and problematic weight loss in the past year compared to those who were not depressed (chi-square test, *p* < 0.01). Although caution should be exercised given that our question on depression was not formally validated, the clustering of depressive symptoms among the same individuals suggests that our single-item measure can be used as an adequate measure of depression.

Measuring social capital is also a complex task. One of the most serious criticisms in relation to social capital is that measurements do not match the theory. Although social capital is a multi-dimensional concept, many studies rely on one-dimensional measures [98]. Few existing instruments measure the various forms of social capital. There is also a lack of consistency among studies. For instance, Mitchell and LaGory [41] measured *bonding *social capital via associational membership, whereas others [99] have used the same measure as an indicator of *bridging* social capital. The value of separating the level of family that exists within social capital has recently been stressed [79]. In this article, an attempt has been made to measure different forms of social relations, distinguishing mainly between social capital within and outside the family. This may be especially important in Russia where forms other than associational membership are important [65, 66]. Although these forms are conceptually different, in reality there is, of course, much overlap between the different forms of social capital [32]. Further, due to the cross-sectional nature of the study, it was not possible to determine the direction of causality. Feeling depressed might be a cause rather than an effect of differences in social capital. A recent study has shown, for example, that the social capital-health relationship is bidirectional: while high levels of social capital promote better health, social capital also depends on health [100]. Based on previous studies and theoretical explanations, however, it is widely recognised and theoretically plausible that social capital has an impact on mental health [16]. A final limitation of this study concerns the regression analyses between age-bridging extra-familial contacts and women’s self-rated depression. Due to the reduced number of cases in the subgroups, the results should be interpreted with some caution. However, the results do give an indication of the groups within which the effects of this kind of social capital are larger (i.e. young women and women with small children). Although intersectional studies are being conducted more frequently to reveal power relations, much work remains to be done, especially in relation to age [59]. Consequently, this topic is important and requires further investigation.

## Conclusions

Social capital can be a mixed blessing for women. Different forms of social relations can lead to different health outcomes, both positive *and* negative. Family is an important resource promoting women’s mental health in Moscow, whereas extra-familial relations across age groups can be mentally distressing. Socialising among women within families often involves an exchange of support, but maintaining ties across age groups outside the family can be stressful due to value disparity, conflict and discrimination. These findings constitute an important contribution from a theoretical perspective because even though the downside of social capital is increasingly being discussed in the literature, it has, until now, rarely been shown empirically. Consequently, this study adds to the few studies on the social capital-depression association that provide empirical evidence for negative mental health aspects of social capital. In conclusion, although social capital can be seen as a valuable resource for mental health, some of its forms can be mentally deleterious to maintain, especially among women.

More research, both quantitative and qualitative, is needed about both sides to social capital – the positive and the negative health aspects of social capital. In relation to this, future research should examine how the social capital-depression association varies by different *forms* of social relations and by different *groups.* It is important to continue distinguishing between different forms of social capital, as they imply different resources and constraints. Researchers should try to identify the forms of social relations that are most valuable as well as most burdening for various groups. When studying social capital and depression, as noted by Vyncke et al. [44], a combination of dimensions, such as gender *and* age, should be analysed. A special focus should be placed on bridging social relations among women, particularly mothers with small children, to better understand the complex association between social capital, gender and depression in Russia and elsewhere.

## Abbreviations

CI, Confidence interval; ISESP, Institute for Social and Economic Studies of Population; OR, Odds ratio; RAS, Russian Academy of Sciences; SCOHOST, Stockholm Centre for Health and Social Change; WHO, World Health Organisation.
