# Erratum to: Social capital—a mixed blessing for women? A cross-sectional study of different forms of social relations and self-rated depression in Moscow

**DOI:** 10.1186/s40359-017-0190-3

**Published:** 2017-06-19

**Authors:** Sara Ferlander, Andrew Stickley, Olga Kislitsyna, Tanya Jukkala, Per Carlson, Ilkka Henrik Mäkinen

**Affiliations:** 1 Stockholm Centre for Health and Social Change (SCOHOST), Department of Sociology, School of Social Sciences, Södertörn University, 141 89 Huddinge, Sweden; 2 European Centre on Health of Societies in Transition (ECOHOST), London School of Hygiene and Tropical Medicine, London, UK; 3 Department of Human Ecology, Graduate School of Medicine, University of Tokyo, Tokyo, Japan; 4 Department of Quality of Life Measurement Problems at the Institute of Economics, Russian Academy of Sciences, Moscow, Russia; 5 Stockholm Centre for Health and Social Change (SCOHOST), Department of Social Work, School of Social Sciences, Södertörn University, Huddinge, Sweden; 6 Department of Sociology, Uppsala University, Uppsala, Sweden

## Erratum

After publication of the original article [1], the author reported an error to the columns depicting the results for “Women (*n* = 678)” in Table 2. The correct version of Table 2 is included in this erratum.

**Table 2:** Depression among respondents aged 18 and over in Moscow

Variables	Men (*n* = 507)				Women (*n* = 678)			
	Model 1		Model 2		Model 1		Model 2	
	OR	95% CI	OR	95% CI	OR	95% CI	OR	95% CI
Age	1.00	0.99–1.01	0.99	0.98–1.00	1.00	0.99–1.01	1.00	0.99–1.01
Educational level								
High	1.00		1.00		1.00		1.00	
Medium	0.89	0.58–1.36	0.78	0.49–1.22	0.91	0.64–1.29	0.85	0.59–1.22
Low	1.04	0.66–1.65	1.02	0.63–1.65	0.75	0.50–1.12	0.74	0.49–1.12
Economic problems								
Few	1.00		1.00		1.00		1.00	
Many	2.34	1.57–3.51^***^	2.49	1.65–3.78^***^	1.48	1.09–2.01^*^	1.52	1.11–2.07^**^
Family relations								
Marital status								
Married/cohabiting	1.00		1.00		1.00		1.00	
Divorced/widowed	1.20	0.70–2.07	1.13	0.64–1.98	1.48	1.02–2.14^*^	1.49	1.03–2.17^*^
Never married	1.03	0.60–1.76	1.10	0.62–1.95	0.96	0.59–1.56	0.94	0.56–1.55
Contact with relatives								
Regular	1.00		1.00		1.00		1.00	
Little	1.62	1.10–2.39^*^	1.51	1.00–2.30	1.45	1.06–1.98^*^	1.57	1.10–2.24^*^
Extra-familial relations								
Contact with friends/acquaintances								
Regular	1.00		1.00		1.00		1.00	
Little	1.63	1.08–2.45^*^	1.44	0.93–2.25	1.12	0.81–1.55	0.97	0.67–1.40
Age-bridging contacts								
Regular	1.00		1.00		1.00		1.00	
Little	0.99	0.67–1.47	0.88	0.58–1.33	0.72	0.52–0.98^*^	0.72	0.51–1.00^*^
Voluntary associations								
Member	1.00		1.00		1.00		1.00	
Non-member	1.07	0.70–1.63	1.07	0.68–1.68	0.96	0.66–1.40	1.04	0.70–1.54

Odds ratios (OR) with 95% confidence intervals (CI) estimated from binary logistic regression. Model 1: age adjusted; Model 2: mutually adjusted

NOTE: Standardised according to the educational-level proportions of the Moscow City population given in the 2002 census for the population over 18 years

^*^ *p* < 0.05 ^**^ *p* < 0.005 ^***^ *p* < 0.001
